# Metacognition and Headache: Which Is the Role in Childhood and Adolescence?

**DOI:** 10.3389/fneur.2017.00650

**Published:** 2017-12-14

**Authors:** Noemi Faedda, Giulia Natalucci, Dario Calderoni, Rita Cerutti, Paola Verdecchia, Vincenzo Guidetti

**Affiliations:** ^1^Department of Paediatric and Child and Adolescent Neuropsychiatry, Sapienza University of Rome, Rome, Italy; ^2^Department of Dynamic and Clinical Psychology, Sapienza University of Rome, Rome, Italy

**Keywords:** metacognition, children, adolescents, headache, comorbidity, theory of mind

## Abstract

Headache, in particular migraine, is one of the most frequent neurological symptoms in children and adolescents and it affects about 60% of children and adolescents all over the world. Headache can affect several areas of child’s functioning, such as school, physical activities, peer, and family relationship. The global and severe burden of this disease requires a multidisciplinary strategy and an effective treatment addressed all of the patient’s needs and based on cutting-edge scientific research. In recent years, research has focused on cognitive factors specifically in functions called metacognitive processes. Metacognition can be defined as the knowledge, beliefs, and cognitive processes involved in monitoring, control, and assessment of cognition. Metacognition seems to be closely related to the ability of theory of mind, the ability to infer, and reason about the mental states of other people in order to predict and explain own behavior. Recent studies found a relationship between metacognitive skills and anxiety, depression, motivation, academic performance, human social interactions, and stress symptoms. This relationship is very interesting for headache treatment, because these factors are the most commonly reported triggers in this disorder and there is a high comorbidity with anxiety and depression in children and adolescents with headache. So, headache and these comorbidities, in particular anxiety and depression, may have in common persistent and maladaptive patterns of thinking which are related to maladaptive metacognitive beliefs. Further research should assess metacognitive processes of children and adolescents with headache in order to increase their ability to control their own cognitive processes and consequently monitor factors which may trigger the attacks.

## Introduction

Recurrent headache and migraine are the most common neurological complaints in children and adolescents and they are recognized as a significant health problem. The prevalence of this neurological disorder in children and adolescents is about 55 and 91% of migraine (1). The global and severe burden of this disease requires a multidisciplinary strategy and an effective treatment addressed all of the patient’s needs and based on cutting-edge scientific research (2, 3). Indeed, primary headache in childhood is often associated with a number of comorbidities, such as asthma, allergies, sleep disorders, emotional and behavioral problems, internalizing disorders, such as depression and anxiety, obesity, etc. (4), and it can negatively affect child and family lives and interfere in daily activities, social interaction, and school performance in combination with psychopathological symptoms (5, 6).

Some studies have focused on possible cognitive deficit in children with headache or migraine. For example, an interesting study realized by Parisi and colleagues (7), investigated differences in intellectual functioning and cognitive profile in 82 children with tension-type headache (TTH) and migraine without aura (MOA), compared with a control group without any disorders. The two groups shown significant differences in the mean total intelligence quotient and verbal intelligence quotient scores (7). Moreover, the authors hypothesized that high frequency of headache attack and its early age at onset are associated with cognitive impairment probably, because in the developmental age the central nervous system is not yet completely mature (7).

Some findings show that the most impaired neuropsychological functions concern selective and divided attention, speed of processing information, and short- and long-term verbal memory (8, 9). Melissa Andréia et al. (10) compared 28 adolescents diagnosed with migraine and 26 subjects without a history of headache after a neuropsychological assessment. They noticed that adolescents with migraine were more affected by distractors during the learning process, had verbal memory and more learning difficulties and shown impairment in recognition and recall (10). These problems may have a connection with learning difficulties sometimes referred by children with migraine or recurrent headache. In contrast with these findings, Riva (11) highlights the greater presence of internalizing problems instead of neuropsychological dysfunctions. According to this hypothesis, Haverkamp et al. (12) conclude that children with migraine were not at risk for impaired cognitive development. So, the authors believe that it should be clarified if specific neuropsychological deficits in patients with migraine are caused by other factors independent from migraine (12).

In recent years, a much studied cognitive function, especially in childhood, is mentalization [including theory of mind (ToM)] or metacognition. These abilities explain or predict behavior based on beliefs, intentions, or feelings attributed to the self or others, mental states, and including thoughts (13). For example, in the last 30 years the role of ToM in autism spectrum disorders (ASD) and other communication impairments has been a prosperous research area (14, 15) and neurobiological studies have discovered interesting brain networks underlying both ASD and ToM (16, 17).

Other psychological and psychiatric disorder in which there could be an impairment or dysfunctions in mentalization ability or ToM are for example ADHD (18, 19), intellectual disability (20), externalizing disorders, also connected with high levels of callous-unemotional (CU) traits (21) and, especially regarding metacognition and metacognitive strategies, learning disabilities (22).

In the following paragraphs will be deepened the role that those psychological constructs have in pediatric headaches and how their role in treatment.

## What is Metacognition?

The concept of metacognition is a relatively recent psychological construct, first introduced by Flavell in 1979. Metacognition can be defined as the knowledge, beliefs, and cognitive processes involved in the monitoring, control, and assessment of cognition (23). These processes involve:

(1) “introspective knowledge about one’s cognitive states and abilities and their operation”;
(2) “the ability to use metacognitive knowledge strategically to achieve goals (strategy regulation)”;
(3) “cognitive monitoring of thoughts (the ability to read one’s own mental state)” (24–27).

It refers to any cognitive or knowledge process that is involved in assessment, monitoring, or controlling cognition and it can be synthesized as “thinking about thinking” (24).

This ability first allows people to identify and recognize mental states in both the self and others. It consents also to think about mental processes, such as cause and effect relationships, emotions, feelings, thoughts, and behaviors and, finally, it helps to understand that ideas are subjective and that people have different point of view and different perspectives on things (28).

Metacognition is linked to other psychological construct such as mentalization and it refers to “the ability to understand ourselves and others in terms of intentional mental states, such as feelings, desires, wishes, attitudes, and goals.”

It is fundamental in our social environment and its acquisition depends on the context of attachment relationships (29). The concept of mentalization contains related constructs, such as empathy, mindfulness, and ToM (30). Indeed, metacognition seems to be closely related to the ability of ToM, the “ability to infer and reason about the mental states of other people in order to predict and explain own behaviour” (31).

Both mentalization and metacognition are included in executive functions, the higher cortical functions. Because of their nature, the more involved cortical areas are the dorsolateral prefrontal cortex, ventromedial prefrontal cortex, medial frontoparietal network, and more in general the prefrontal areas and the frontal lobe (29, 32).

It is commonly assumed that the acquisition of fully developed explicit concept of mental states starts around age 4, period in which children start to control standard verbal tasks necessary for the identification of “false beliefs to other agents” (33, 34). The evaluation of mentalization or ToM usually consists of tasks that exhort participants to infer others’ mental states, for example, completing stories embody the protagonist’s point of view (35). These tests are also called “false believes task” and they involve first- or second-order false beliefs according to the task difficulty. While the first-order beliefs refer to assumptions made about another person’s beliefs, the second-order beliefs deal with another person’s assumptions about a third individual’s belief (36). One most famous and commonly used false belief task, developed by Wimmer and Perner first (37) and implemented later by Baron-Cohen et al. (38), is the Sally–Anne test, in which there are two story characters with two different belief states with respect to a hidden object.

## What is the Role of Metacognition in Headache?

As widely described above, psychological constructs as metacognition and ToM are broadly studied and evaluated in many psychiatric diseases. However, there are not numerous studies that explore the relationship between headache, in particular migraine and metacognition especially in children and adolescents. There are some studies (conducted whit adult patients) that find a connection between somatoform pain disease and deficit in ToM. For example, Zunhammer and colleagues (39) found that chronic somatoform pain patients (included headache/migraine suffering) had more difficulties in their mentalizing abilities compared to healthy controls. Moreover, they evidenced in their patient significant impairment in emotional awareness.

According to this study, Preis et al. (40) reported impairment in affective ToM in patients with somatoform disease related to people without any disease, but they do not identify significant differences in cognitive ToM between the two groups. A study conducted by Petolicchio and colleagues (41) involved 57 patients with chronic migraine, with or without medication-overuse headache (MOH). They suggest that there could be a possible connection between the chronicization of migraine and a low mentalizing level.

While some studies have demonstrated deficit in ToM in children with neurological disorders such as epilepsy (42), no one have yet demonstrated the same thing in children with headache or migraine. Only one study has considered the possible impairment in mentalization ability in children with headache and epilepsy. La Grutta and colleagues (43) enrolled 65 children from 7 to 11 (48 males and 27 females) suffering from epilepsy and primary headache. For the evaluation of mentalization quality, they use semi-structured interview on dreams and to assessment the quality of the psychological suffering they used the drawing stories technique. Their results suggest that psychological suffering due to body sickness can affect some mental representation and way to think.

Since headache in pediatric age is widespread, complex, and often in comorbidities with other disease (psychiatric, neurological, etc.), deepening possible relationship with mentalization could be helpful to children to improve social skills and their quality of life.

## Headache, Comorbidities, and Bad Life Events

Although some studies found inconsistent results showing that the majority of children and adolescents with chronic daily headache do not have a psychiatric comorbidity (44) and suggesting that it is difficult to clearly differentiate some migraine features from psychiatric diagnoses with the current screening tools available (45), the majority of studies in this field identified a high presence of psychological comorbidities in children and adolescents with headache (4).

A cross-sectional, population-based study, called Young-HUNT1 was conducted in Norway from 1995 to 1997. In this study, 4,872 adolescents (12–17 years) were interviewed about their headache complaints and completed a comprehensive questionnaire that included assessment of symptoms of anxiety and depression and behavioral problems. The authors found higher levels of symptoms of anxiety and depression in adolescents aged 12–14 years with recurrent headache, while in adolescents aged 15–17 years with higher levels of anxiety and depression were associated with all diagnosis of headache (46).

A recent study confirmed not only that children and adolescents with MoA have higher anxiety levels than a control healthy group but also it shows differences with respect to security of attachment to both mother and father between two groups. Thus, the authors suggested that “the migraine-anxiety association would be at least partially mediated by attachment security” (47).

Headache would seem to be associated with bad life events, in particular with childhood abuse and neglect (48). Tietjen et al. (49) assessed the prevalence of childhood maltreatment and adult revictimization in patient with migraine taking in account several factors such as the presence of anxiety or depression and sociodemographic factors. They found that: “reports of childhood maltreatment, especially emotional abuse and neglect, were prevalent in outpatients with migraine and all types of childhood abuse and neglect were strongly associated with remote and current depression and anxiety, and the relationship strengthens with an increasing number of maltreatment types” (49).

Some studies found that younger with chronic pain and comorbid depression are at increased risk of thinking about and attempting suicide (50), in particular it was shown a high comorbidity suicidal risk in adolescents with chronic daily headache (51).

Moreover, several researches have found that children and adolescents with migraine compared with a healthy sample show difficulty in expressing emotional states (52), displaying a higher level of alexithymic characteristics (53, 54).

Alexithymia refers to a “personality construct that implies a poor ability to identify and describe feelings, a reduced imagination, and a concrete, externally-oriented way of thinking” (53), and it would seem to be associated with metacognition skills, understood as the beliefs and attitudes of an individual about cognitive events like thoughts, emotions, memories, feelings, and other perceptual forces (55, 56).

So, headache and these comorbidities, in particular anxiety and depression, may have in common several persistent and maladaptive patterns of thinking which are related to maladaptive metacognitive beliefs (57).

According to this metacognition theory and in the light of this scientific evidences that report a greater presence of stressful life situations in patients with headache, the use of metacognition therapy, and mindfulness practices could be effective means of swiftly modifying cognitive events, such as emotions, thoughts, and bad memories.

## The Role of Metacognition, Cognitive Behavioral Therapy (CBT), and Mindfulness in the Treatment of Psychopathological Comorbidities in Headache

Non-pharmacological therapy, in particular behavioral strategies seem to be as effective as pharmacological treatment for headache management (3, 58, 59).

Ideal candidates for behavioral intervention seem to be patients with chronic pain and clinical depression or anxiety or individuals with reduced ability to manage triggers (e.g., stress) or with other significant psychological problems (e.g., history of abuse/maltreatment) (60–62).

Behavioral therapy consists of three main components (63):

1. Treatment adherence;
2. Adjustment of lifestyle management;
3. Psychological intervention.

In particular, psychological interventions include (64):

- Relaxation skills;
- Biofeedback;
- Cognitive behavioral therapy.

Relaxation skills, such as autogenic phrases, guided imagery (GI), self-hypnosis, diaphragmatic breathing, and progressive muscle relaxation (PMR), seem to be as effective as pharmacological treatment in children, adolescents, and adult, improving the clinical features, such as frequency, intensity, and duration of headache (65, 66).

Cognitive behavioral therapy consists mainly of cognitive and behavioral strategies with the aim to change patient exaggerated or irrational thought patterns, dysfunctional negative emotions, interpretations of events, and maladaptive behavioral patterns of responding to stressors or events, enhancing patient’s ability to cope with the pain, and to reduce headache-related distress (3, 67).

Among CBTs, the most effective treatment for the management of childhood abuse or maltreatment seems to be the trauma-focused CBT (TF-CBT) (68, 69).

Trauma-focused cognitive behavioral therapy includes cognitive behavioral, family, humanistic, and trauma-sensitive interventions and techniques. In this therapy, “children and their parents are taught skills to help them elaborate thoughts and feelings related to traumatic life events and manage and resolve distressing thoughts, feelings, and behaviors related to traumatic life events” (70).

Cognitive behavioral therapy is strictly related to metacognition and mindfulness practices: “metacognitive therapy is based on the principle that worry and rumination are universal processes leading to emotional disorder” (71).

These processes are associated with inefficient self-regulation, coping strategies, and erroneous thinking and so mindfulness tries to change the individual’s perspective toward own relationship, thoughts and feelings, observing what occurs, with a special focus on the contents of inner experience, without evaluating or judging (71).

Several researches found that mindfulness can benefit adults with headache (72), but very few studies have been conducted with children and adolescents.

Hesse et al. (73) conducted a pilot clinical trial with 20 adolescent females with recurrent headaches, showing that although mindfulness-based intervention did not report decreased frequency or severity of headache, it had a beneficial effect for depression, quality of life, and acceptance of pain.

Mindfulness seems to play an important role in cognitive and behavioral reactions to daily pain. Petter et al. (74) found that “mindfulness is negatively associated with typical pain intensity and pain catastrophizing, and it is a unique and nonredundant predictor of how much pain interferes in the lives of adolescents.”

Przekop et al. compared a multimodal (“osteopathic manipulative treatments, mindfulness, and qi gong”) and pharmacologic (amitriptyline or gabapentin) treatment in children and adolescents with chronic TTH, showing that the multimodal treatment was significant in reducing headache frequency and pain in increasing participants’ quality of life, reducing the physical and emotional problems reported by patients (75).

Kemper et al. (76) conducted a study on adolescents who had multi-year histories of recurrent headaches, showing that the depression was positively associated with stress, anxiety, and sleep disturbances and negatively associated with mindfulness, self-compassion, and resilience.

Resilience also, seems to play a role in pain self-efficacy and acceptance in patients with chronic pain. Resilience refers to “a class of phenomena characterized by good outcomes despite serious threats to adaptation or development” (77). In the context of pediatric chronic pain, a child’s resilience might be conceptualized as “the personal resources and effective responding that protect from dysfunction, lead to adaption, or result in well-being and growth” (78).

Sturgeon and Zautra (79) proposed the process of resilience to pain like a multimodal paradigm for understanding pain and pain coping, that can “identify the traits and mechanisms underlying the sustainability of a good life and recovery from distress.”

In particular, two processes of resilience have been considered in children with chronic pain: *pain acceptance* and *pain self-efficacy*. Kalapurakkel et al. (80) showed that these two processes were negatively related with the disability and the depressive symptoms and positively related with a school functioning. In particular, pain acceptance seems to have a greater association with less depressive symptoms and better school functioning, while pain self-efficacy seems to have a greater association with less functional disability (80).

## Conclusion

Further studies and evidences are needed, but the use of metacognitive strategies and mindfulness practice in children and adolescents with headaches could be a preventative factor for the development or strengthening of psychiatric comorbidities, especially in children with chronic and recurrent headache who are at increased risk for psychiatric and behavioral comorbidities.

## Author Contributions

VG, NF, and GN conceived and designed the study and they are responsible for data acquisition. DC, RC, and PV were responsible for critical revision of this manuscript. All authors approved the final version of this manuscript.

## Conflict of Interest Statement

The authors declare that the research was conducted in the absence of any commercial or financial relationships that could be construed as a potential conflict of interest.

## References

[1] Wöber-Bingöl C. Epidemiology of migraine and headache in children and adolescents. Curr Pain Headache Rep (2013) 17(6):341. 10.1007/s11916-013-0341-z 23700075

[2] Gaul C Visscher CM Bhola R Sorbi MJ Galli F Rasmussen AV Team players against headache: multidisciplinary treatment of primary headaches and medication overuse headache. J Headache Pain (2011) 12(5):511–9. 10.1007/s10194-011-0364-y 21779789 PMC3173636

[3] Faedda N Cerutti R Verdecchia P Migliorini D Arruda M Guidetti V Behavioral management of headache in children and adolescents. J Headache Pain (2016) 17(1):80 10.1186/s10194-016-0671-4 27596923 PMC5011470

[4] Bellini B Arruda M Cescut A Saulle C Persico A Carotenuto M Headache and comorbidity in children and adolescents. J Headache Pain (2013) 24(14):79. 10.1186/1129-2377-14-79 24063537 PMC3849985

[5] Hershey AD. Pediatric headache. Continuum (Minneap Minn) (2015) 21(4 Headache):1132–45. 10.1212/CON.0000000000000197 26252596

[6] Dyb G Stensland S Zwart JA. Psychiatric comorbidity in childhood and adolescence headache. Curr Pain Headache Rep (2015) 19(3):5. 10.1007/s11916-015-0479-y 25754599 PMC4353875

[7] Parisi P Verrotti A Paolino MC Urbano A Bernabucci M Castaldo R Headache and cognitive profile in children: a cross-sectional controlled study. J Headache Pain (2010) 11(1):45–51. 10.1007/s10194-009-0165-8 19841863 PMC3452186

[8] Villa T Correa Moutran A Sobirai Diaz L Pereira Pinto MM Carvalho FA Gabbai AA Visual attention in children with migraine: a controlled comparative study. Cephalalgia (2009) 29:631–4. 10.1111/j.1468-2982.2008.01767.x 19187339

[9] Waldie K Hausmann M Milne B Poulton R. Migraine and cognitive function: a life-course study. Neurology (2002) 59:904–8. 10.1212/WNL.59.6.904 12297575

[10] Melissa Andréia CS Ana Carolina de Almeida P Leonardo Cruz de S Rodrigo Santiago G Antônio Lúcio T Cognitive functioning in adolescents with migraine. Dement Neuropsychol (2016) 10(1):47–51. 10.1590/s1980-57642016dn10100009 29213431 PMC5674914

[11] Riva D Aggio F Vago C Nichelli F Andreucci E Paruta N Cognitive and behavioural effects of migraine in childhood and adolescence. Cephalalgia (2006) 26(5):596–603. 10.1111/j.1468-2982.2006.01072.x 16674769

[12] Haverkamp F Hönscheid A Müller-Sinik K Cognitive development in children with migraine and their healthy unaffected siblings F. Headache (2002) 42:776–9. 10.1046/j.1526-4610.2002.02179.x 12390640

[13] Stonnington CM Locke DE Hsu CH Ritenbaugh C Lane RD. Somatization is associated with deficits in affective theory of mind. J Psychosom Res (2013) 74(6):479–85. 10.1016/j.jpsychores.2013.04.004 23731744

[14] Schuwerk T Vuori M Sodian B. Implicit and explicit theory of mind reasoning in autism spectrum disorders: the impact of experience. Autism (2015) 19(4):459–68. 10.1177/1362361314526004 24627427

[15] Angus DJ de Rosnay M Lunenburg P Meerum Terwogt M Begeer S. Limitations in social anticipation are independent of imaginative and theory of mind abilities in children with autism but not in typically developing children. Autism (2015) 19(5):604–12. 10.1177/1362361314537911 24923896

[16] Cheng W Rolls ET Gu H Zhang J Feng J. Autism: reduced connectivity between cortical areas involved in face expression, theory of mind, and the sense of self. Brain (2015) 138(Pt 5):1382–93. 10.1093/brain/awv051 25795704 PMC4407191

[17] O’Nions E Sebastian CL McCrory E Chantiluke K Happé F Viding E. Neural bases of theory of mind in children with autism spectrum disorders and children with conduct problems and callous-unemotional traits. Dev Sci (2014) 17(5):786–96. 10.1111/desc.12167 24636205 PMC4316185

[18] Maoz H Tsviban L Gvirts HZ Shamay-Tsoory SG Levkovitz Y Watemberg N Stimulants improve theory of mind in children with attention deficit/hyperactivity disorder. J Psychopharmacol (2014) 28(3):212–9. 10.1177/0269881113492030 23761389

[19] Caillies S Bertot V Motte J Raynaud C Abely M. Social cognition in ADHD: irony understanding and recursive theory of mind. Res Dev Disabil (2014) 35(11):3191–8. 10.1016/j.ridd.2014.08.002 25155741

[20] Fiasse C Nader-Grosbois N. Perceived social acceptance, theory of mind and social adjustment in children with intellectual disabilities. Res Dev Disabil (2012) 33(6):1871–80. 10.1016/j.ridd.2012.05.017 22705455

[21] Song JH Waller R Hyde LW Olson SL Early callous-unemotional behavior, theory-of-mind, and a fearful/inhibited temperament predict externalizing problems in middle and late childhood. J Abnorm Child Psychol (2016) 44(6):1205–15. 10.1007/s10802-015-0099-3 26582182 PMC4937620

[22] Nicolielo-Carrilho AP Hage SRV. Metacognitive reading strategies of children with learning disabilities. Codas (2017) 29(3):e20160091. 10.1590/2317-1782/20172016091 28538827

[23] Wells A Emotional Disorders and Metacognition: Innovative Cognitive Therapy. Chichester, UK: Wiley (2000). 252 p.

[24] Bacow TL Pincus DB Ehrenreich JT Brody LR The metacognitions questionnaire for children: development and validation in a clinical sample of children and adolescents with anxiety disorders. J Anxiety Disord (2009) 23(6):727–36. 10.1016/j.janxdis.2009.02.013 19362445

[25] Brown AL Bransford JD Ferrera RA Campione JC Learning remembering and understanding. In: Flavell JH Markman EM, editors. Handbook of Child Psychology: Cognitive Development. (Vol. 1), New York: Wiley Pressley (1983). p. 77–166.

[26] Pressley M Borkowki JG O’Sullivan J Children’s metamemory and the teaching of memory strategies. In: Yussen SR, editor. Metacognition, Cognition and Human Performance. (Vol. 1), New York: Press Wellman (1985) 115–53.

[27] Wellman HM The Origins of Metacognition. Metacognition Cognition and Human Performance. (Vol. 1). New York: Academic Press (1985). p. 1–31.

[28] Dimaggio G Lysaker PH. Metacognition and mentalizing in the psychotherapy of patients with psychosis and personality disorders. J Clin Psychol (2015) 71(2):117–24. 10.1002/jclp.22147 25557317

[29] Luyten P Fonagy P The neurobiology of mentalizing. Personal Disord (2015) 6(4):366–79. 10.1037/per0000117 26436580

[30] Fonagy P Attachment, Personality Disorder and Its Psychological Treatment. Available from: https://www.iasa-dmm.org/images/uploads/Peter%20Fonagy-%20Attachment,%20Personality%20Dis%20and%20its%20psychological%20tx.pdf

[31] Langdon R Still M Connors MH Ward PB Catts SV. Theory of mind in early psychosis. Early Interv Psychiatry (2014) 8(3):286–90. 10.1111/eip.12072 23837736

[32] Ardila A. Development of metacognitive and emotional executive functions in children. Appl Neuropsychol Child (2013) 2(2):82–7. 10.1080/21622965.2013.748388 23848243

[33] Rakoczy H Bergfeld D Schwarz I Fizke E. Explicit theory of mind is even more unified than previously assumed: belief ascription and understanding aspectuality emerge together in development. Child Dev (2015) 86(2):486–502. 10.1111/cdev.12311 25345545

[34] Baillargeon R Scott RM He Z False-belief understanding in infants. Trends Cogn Sci (2010) 14(3):110–8. 10.1016/j.tics.2009.12.006 20106714 PMC2930901

[35] Moran JM Young LL Saxe R Lee SM O’Young D Mavros PL Impaired theory of mind for moral judgment in high-functioning autism. Proc Natl Acad Sci U S A (2011) 108(7):2688–92. 10.1073/pnas.1011734108 21282628 PMC3041087

[36] Boucher J Putting theory of mind in its place: psychological explanations of the socio-emotional-communicative impairments in autistic spectrum disorder. Autism (2012) 16:226–46. 10.1177/1362361311430403 22297199

[37] Wimmer H Perner J Beliefs about beliefs: representation and constraining function of wrong beliefs in young children’s understanding of deception. Cognition (1983) 13:103–28. 10.1016/0010-0277(83)90004-5 6681741

[38] Baron-Cohen S Leslie AM Frith U Does the autistic child have a “theory of mind”? Cognition (1985) 21(1):37–46. 10.1016/0010-0277(85)90022-8 2934210

[39] Zunhammer M Halski A Eichhammer P Busch V. Theory of mind and emotional awareness in chronic somatoform pain patients. PLoS One (2015) 10(10):e0140016. 10.1371/journal.pone.0140016 26445110 PMC4596852

[40] Preis MA Golm D Kröner-Herwig B Barke A Examining differences in cognitive and affective theory of mind between persons with high and low extent of somatic symptoms: an experimental study. BMC Psychiatry (2017) 17(1):200 10.1186/s12888-017-1360-9 28558727 PMC5450064

[41] Petolicchio B Squitieri M Viganò A Toscano M Sirolli A Aielli S Psychodynamic functioning in chronic headache patients: a short term psychodynamic psychotherapy (STPP) study. J Headache Pain (2015) 16(Suppl 1):A105 10.1186/1129-2377-16-S1-A105 28132283 PMC4715059

[42] Stewart E Catroppa C Lah S. Theory of mind in patients with epilepsy: a systematic review and meta-analysis. Neuropsychol Rev (2016) 26(1):3–24. 10.1007/s11065-015-9313-x 26797753

[43] La Grutta S Lo Baido R Schiera G Trombini E Trombini G Sarno L Symbolic function explored in children with epilepsy and headache. Minerva Pediatr (2007) 59(6):745–54. 17978783

[44] Gelfand AA. Psychiatric comorbidity and paediatric migraine: examining the evidence. Curr Opin Neurol (2015) 28(3):261–4. 10.1097/WCO.0000000000000192 25923126

[45] Qubty W Gelfand AA. Psychological and behavioral issues in the management of migraine in children and adolescents. Curr Pain Headache Rep (2016) 20(12):69. 10.1007/s11916-016-0597-1 27896587

[46] Blaauw BA Dyb G Hagen K Holmen TL Linde M Wentzel-Larsen T Anxiety, depression and behavioral problems among adolescents with recurrent headache: the Young-HUNT study. J Headache Pain (2014) 15:38. 10.1186/1129-2377-15-38 24925252 PMC4062897

[47] Williams R Leone L Faedda N Natalucci G Bellini B Salvi E The role of attachment insecurity in the emergence of anxiety symptoms in children and adolescents with migraine: an empirical study. J Headache Pain (2017) 18(1):62 10.1186/s10194-017-0769-3 28560542 PMC5449355

[48] Tietjen GE Buse DC Fanning KM Serrano D Reed ML Lipton RB Recalled maltreatment, migraine, and tension-type headache: results of the AMPP study. Neurology (2015) 84(2):132–40. 10.1212/WNL.0000000000001120 25540306 PMC4336089

[49] Tietjen GE Brandes JL Peterlin BL Eloff A Dafer RM Stein MR Childhood maltreatment and migraine (part I). Prevalence and adult revictimization: a multicenter headache clinic survey. Headache (2010) 50(1):20–31. 10.1111/j.1526-4610.2009.01556.x 19845782

[50] van Tilburg MA Spence NJ Whitehead WE Bangdiwala S Goldston DB. Chronic pain in adolescents is associated with suicidal thoughts and behaviors. J Pain (2011) 12(10):1032–9. 10.1016/j.jpain.2011.03.004 21684217 PMC3178682

[51] Wang SJ Juang KD Fuh JL Lu SR. Psychiatric comorbidity and suicide risk in adolescents with chronic daily headache. Neurology (2007) 68(18):1468–73. 10.1212/01.wnl.0000260607.90634.d6 17470748

[52] Mazzone L Vitiello B Incorpora G Mazzone D. Behavioural and temperamental characteristics of children and adolescents suffering from primary headache. Cephalalgia (2006) 26(2):194–201. 10.1111/j.1468-2982.2005.01015.x 16426275

[53] Cerutti R Valastro C Tarantino S Valeriani M Faedda N Spensieri V Alexithymia and psychopathological symptoms in adolescent outpatients and mothers suffering from migraines: a case control study. J Headache Pain (2016) 17:39. 10.1186/s10194-016-0640-y 27093870 PMC4837193

[54] Gatta M Canetta E Zordan M Spoto A Ferruzza E Manco I Alexithymia in juvenile primary headache sufferers: a pilot study. J Headache Pain (2011) 12(1):71–80. 10.1007/s10194-010-0248-6 20730593 PMC3072508

[55] Saed O Purehsan S Aslani J Zargar M The role of thought suppression, meta-cognitive factors and negative emotions in prediction of substance dependency disorder. Res Addict (2011) 5(18):69–84.

[56] Babaei S Gharechahi M Hatami Z Ranjbar Varandi S. Metacognition and body image in predicting alexithymia in substance abusers. Int J High Risk Behav Addict (2015) 4(3):e25775. 10.5812/ijhrba.25775 26495262 PMC4609502

[57] Cook SA Salomon P Dunn G Fisher P Measuring metacognition in cancer: validation of the metacognitions questionnaire 30 (MCQ-30). PLoS One (2014) 9(9):e107302 10.1371/journal.pone.0107302 25215527 PMC4162595

[58] Damen L Bruijn J Koes BW Berger MY Passchier J Verhagen AP Prophylactic treatment of migraine in children. Part 1. A systematic review of non-pharmacological trials. Cephalalgia (2006) 26(4):373–83. 10.1111/j.1468-2982.2005.01046.x 16556238

[59] Weeks RE. Application of behavioral therapies in adult and adolescent patients with chronic migraine. Neurol Sci (2013) 34(1):S11–7. 10.1007/s10072-013-1360-6 23695037

[60] Campbell JK Penzien D Wall EM Evidence-Based Guidelines for Migraine Headache: Behavioral and Physical Treatments. American Academy of Neurology (2000). Available from: http://tools.aan.com/professionals/practice/pdfs/gl0089.pdf

[61] Robbins M Grosberg BM Lipton R Headache. In: Gross RA Mink JW, editors. Neurology in Practice. NY, USA: Wiley-Blackwell (2013). 336 p.

[62] Rathier L Roth J A biobehavioral approach to headache management. Rhode Island Med J (2015) 98(2):26–8. 25649094

[63] Winner P Migraine. Diagnosis and treatment. In: Hershey AD Powers SW Winner P Kabbouche MA, editors. Pediatric Headaches in Clinical Practice. Oxford: John Wiley & Sons (2009). p. 83–95.

[64] Sieberg CB Huguet A von Baeyer CL Seshia S. Psychological interventions for headache in children and adolescents. Can J Neurol Sci (2012) 39(1):26–34. 10.1017/S0317167100012646 22384492

[65] Sieberg RGJ McGrath PJ. Psychological treatments for migraine. Biomed Pharmacother (1996) 50(2):58–63. 10.1016/0753-3322(96)84714-7 8761710

[66] Engel JM Rapoff MA Pressman AR. Long-term follow-up of relaxation training for pediatric headache disorders. Headache (1992) 32(3):152–6. 10.1111/j.1526-4610.1992.hed3203152.x 1563948

[67] Kabbouche MA Gilman DK. Management of migraine in adolescents. Neuropsychiatr Dis Treat (2008) 4(3):535–48. 10.2147/NDT.S495 18830400 PMC2526375

[68] Martsolf DS Draucker CB. Psychotherapy approaches for adult survivors of childhood sexual abuse: an integrative review of outcomes research. Issues Ment Health Nurs (2005) 26(8):801–25. 10.1080/01612840500184012 16203637

[69] Leenarts LE Diehle J Doreleijers TA Jansma EP Lindauer RJ. Evidence-based treatments for children with trauma-related psychopathology as a result of childhood maltreatment: a systematic review. Eur Child Adolesc Psychiatry (2013) 22(5):269–83. 10.1007/s00787-012-0367-5 23266844

[70] Tietjen GE Buse DC Collins SA. Childhood maltreatment in the migraine patient. Curr Treat Options Neurol (2016) 18(7):31. 10.1007/s11940-016-0415-4 27194425

[71] Fisher P Wells A Metacognitive therapy. The CBT distinctive features series. Eur J Psychol (2009) 4:146–9.

[72] Grazzi L Sansone E Raggi A D’Amico D De Giorgio A Leonardi M Mindfulness and pharmacological prophylaxis after withdrawal from medication overuse in patients with chronic migraine: an effectiveness trial with a one-year follow-up. J Headache Pain (2017) 18(1):15. 10.1186/s10194-017-0728-z 28161874 PMC5292107

[73] Hesse T Holmes LG Kennedy-Overfelt V Kerr LM Giles LL Mindfulness-based intervention for adolescents with recurrent headaches: a pilot feasibility study. Evid Based Complement Alternat Med (2015) 2015:508958 10.1155/2015/508958 26798398 PMC4700163

[74] Petter M Chambers CT McGrath PJ Dick BD. The role of trait mindfulness in the pain experience of adolescents. J Pain (2013) 14(12):1709–18. 10.1016/j.jpain.2013.08.015 24290451

[75] Przekop P Przekop A Haviland MG. Multimodal compared to pharmacologic treatments for chronic tension-type headache in adolescents. J Bodyw Mov Ther (2016) 20(4):715–21. 10.1016/j.jbmt.2015.02.003 27814849

[76] Kemper KJ Heyer G Pakalnis A Binkley PF What factors contribute to headache-related disability in teens? Pediatr Neurol (2016) 56:48–54. 10.1016/j.pediatrneurol.2015.10.024 26810775 PMC5248515

[77] Masten AS. Ordinary magic. Resilience processes in development. Am Psychol (2001) 56(3):227–38. 10.1037/0003-066X.56.3.227 11315249

[78] Cousins LA Kalapurakkel S Cohen LL Simons LE. Topical review: resilience resources and mechanisms in pediatric chronic pain. J Pediatr Psychol (2015) 40(9):840–5. 10.1093/jpepsy/jsv037 25979085 PMC4643616

[79] Sturgeon JA Zautra AJ. Resilience: a new paradigm for adaptation to chronic pain. Curr Pain Headache Rep (2010) 14(2):105–12. 10.1007/s11916-010-0095-9 20425199 PMC4899321

[80] Kalapurakkel S Carpino EA Lebel A Simons LE “Pain can’t stop me”: examining pain self-efficacy and acceptance as resilience processes among youth with chronic headache. J Pediatr Psychol (2015) 40(9):926–33. 10.1093/jpepsy/jsu091 25324532 PMC4592326

